# The Associations between Social Support, Health-Related Behaviors, Socioeconomic Status and Depression in Medical Students

**DOI:** 10.4178/epih/e2010009

**Published:** 2010-11-24

**Authors:** Yoolwon Jeong, Jin Young Kim, Jae Seon Ryu, Ko eun Lee, Eun Hee Ha, Hyesook Park

**Affiliations:** 1 Department of Preventive Medicine, Ewha Womans University School of Medicine, Seoul, Korea.; 2 College of Medicine, Ewha Womans University, Seoul, Korea.

**Keywords:** Depression, Medical students, Social support, Health-related behavior, Socioeconomic status

## Abstract

**OBJECTIVES:**

The objective of this study was to estimate the prevalence of depression in medical students and to evaluate whether interpersonal social support, health-related behaviors, and socio-economic factors were associated with depression in medical students.

**METHODS:**

The subjects in this study were 120 medical students in Seoul, Korea who were surveyed in September, 2008. The subjects were all women and over the age of 20. Their age, body mass index (BMI), quality of sleep, diet, household income, smoking, alcohol consumption, exercise levels, and self-reported health status were surveyed. The degree of perceived social support was measured using the interpersonal support evaluation list (ISEL). Depression was evaluated using the center for epidemiology studies depression scale (CES-D).

**RESULTS:**

The mean CES-D score was 14.1±8.6 and 37.1% of the participants appeared to suffer from depression. Low levels of perceived interpersonal support increased the risk of depression by more than 10 times and having higher household income did not necessarily decrease the risk of depression.

**CONCLUSION:**

Medical students have a relatively high level of depression. Efforts should be made to encourage social support in order to promote mental health in medical students.

## INTRODUCTION

Medical training is a time when medical students undergo a great deal of mental and emotional distress. The psychological status of medical students has become a source of concern as many previous studies have revealed higher levels of depression among medical students in comparison to the general population [1-6]. A higher prevalence rate of depression and suicide among physicians compared to other professions is assumed to have its roots in medical schools and may reflect the effects of untreated depression [7-9]. Therefore, medical students' mental health is not only an issue for the individuals who are affected, but also for the patients for whom they will provide care.

Identifying the modifiable risk factors which affect medial students' depression and the mental distress which they undergo is an important process in the promotion of medical students' mental well-being. Medical students incorporate various health behavior changes in order to cope with the academic burden and stress of medical schools, such as sleep deprivation, an irregular diet, and substance abuse, such as the excessive consumption of alcohol and smoking [10-15]. They also experience the degeneration of their social relationships due to the demanding schedules of medical training [10, 16]. As the ability to maintain supportive relationships is known to have a positive effect when dealing with stress and alleviating depressive symptoms, there is a possibility that this disintegration of social relationships may also be associated with depression among medical students [10, 16-20]. The economic status of an individual or household has frequently been reported to be a risk factor for depression among the general population in numerous previous studies [21-24]. As medical schools impose a great economic burden on students and their families, their economic status should be considered as a possible factor associated with their depression.

Although the issue has great significance, depression in medical students and its associated factors have not yet been thoroughly studied in Korea. The objective of our study was to estimate the prevalence of depression in medical students and evaluate whether perceived interpersonal social support, health-related behaviors and socio-economic factors were associated with depression in medical students.

## MATERIALS AND METHODS

### Subjects and study design

The subjects of this study were all of the 147 first and second year graduate students from a medical school in Seoul and they were surveyed in September 2008. The subjects were all women and over the age of 20. The response rate was 81.6%, with a total of 120 responses collected. We excluded the responses of 31 subjects who had not answered at least one of the items on the interpersonal support evaluation list or the depression scale. In order to assess whether those who were excluded were inherently different from those who were included in the analysis, we carried out a separate analysis in which we found no significant differences in terms of factors of interest such as quality of sleep, diet, smoking, alcohol consumption, exercise, and self-reported health status. Therefore, 89 subjects were included in the final analyses.

### Survey and measures

A survey questionnaire was constructed in order to evaluate the severity of depression, the level of perceived interpersonal social support, health-related behaviors, and the socioeconomic status of the students. Their age, body mass index (BMI), sleep quality, diet, household income, smoking, alcohol consumption, exercise, and self-reported health status were surveyed. In response to the survey question "Do you have meals regularly?", the participants were asked to indicate whether the answer was "regular", "only skips breakfast" or "irregular". In the analysis, "only skips breakfast" and "irregular" were both considered under the heading "irregular". In response to the question, "How often do you exercise?", the answers that could be selected were "never", "1-2 times a week" or "more than 3 times a week". In the analysis, the answers were dichotomized by grouping "1-2 times a week" and "more than 3 times a week" together, under the heading "yes". In response to the question "Do you smoke?", the available answers were "never", "stopped smoking" or "currently smoking". In the analysis, the answers were dichotomized as either "yes" or "no", in which the "yes" group contained both current and past smokers. In response to the question, "How many times did you drink alcohol during the past month?", the respondents could answer as "none", "fewer than 3 times" or "more than 4 times". In the analysis, the answers were dichotomized by grouping those who checked "fewer than 3 times" and "more than 4 times" into the same category group ("yes"). In response to the question, "What is the average income of your household?", the answers to choose from were "less than 1,000 thousand won", "more than 1,000-less than 2,000", "more than 2,000-less than 3,000", "more than 3,000-less than 5,000" and "more than 5,000". The participants' self-reported health status was evaluated by asking "How would you evaluate your current health status?". The participants decided whether their own health status was "good", "neither good nor poor" or "poor".

We used the interpersonal support evaluation list (ISEL) in order to estimate the degree of perceived social support among study subjects. The ISEL is a 40-item evaluation scale which measures the perceived levels of social support. It comprises of four subscales reflecting separate support functions: (1) appraisal (availability of someone to talk to about one's problems), (2) belonging (availability of people to do things with), (3) tangibility (the availability of material aid), and (4) self-esteem (positive comparison of oneself with others). All of the items were assessed using a four-point scale ranging from definitely false (0) to definitely true (3). Although higher total scores indicate a more positive perception of the availability of social support, a cut-off value for the optimum level of perceived social support has not been widely established. We used the median value in order to compare the groups who perceived low and high levels of interpersonal support groups. The internal consistency of the scale has been reported to be between α=0.88 and α=0.90, with a six-month test-retest reliability level of 0.74 [25, 26].

The degree to which depressive symptoms were present was evaluated using the center for epidemiology studies depression scale (CES-D). The CES-D is a self-report scale comprising 20 items which measures the major dimensions of depression [27]. The frequency of each item is scored on a four-point scale ranging from 0 (rarely or none of the time) to 3 (most or all of the time). Higher scores indicate more depressive responses and a score of 16 or greater is generally considered to be indicative of a depressive disorder. It has been reported that with the frequently used cut-off of 16, the sensitivity of the scale for major depression is 100%, and the level of specificity is 88% [28]. The reliability of the scale has been reported to be over α=0.85 [28, 29].

### Statistical analysis

The Wilcoxon rank sum test and the Kruskal-Wallis test were used to compare the CES-D scores according to the factors of interest. The Chi-square test and Fisher's exact test were used in order to compare the rates of depression. Bivariate and multivariate logistic regressions were carried out in order to evaluate the association between various factors and depression. All statistical analyses were conducted using SAS version 9.1 (SAS Inc., Cary, NC). A p value of <0.05 was considered to be statistically significant.

## RESULTS

The mean CES-D score was 14.1±8.6. The ISEL scores ranged from 22 to 108, with a median of 91.0. Perceived levels of interpersonal support, quality of sleep, diet, and self-reported health status were shown to have significant association with the CES-D scores (Table 1). The mean CES-D score in the group who perceived low levels of interpersonal support was 18.4±7.8, which was higher than the mean score of the group who perceived higher levels of support (p<0.01). Students who had poor quality of sleep and an irregular diet also had significantly higher CES-D scores (16.9±8.5 and 16.6±8.3, respectively). The mean CES-D score of those who had reported their health status to be 'poor' was 19.3±8.1, which was higher than those who had reported their status as 'neither good nor poor' or 'good' (p=0.02).

When the prevalence of depression was evaluated using a cut-off value of 16, 33 (37.1%) of the students suffered from depression (Table 2). The prevalence of depression among students who perceived low levels of interpersonal support and had poor quality of sleep were significantly higher than those with higher level of support (p<0.001) and good quality of sleep (p<0.01), respectively. The prevalence of depression were both 50.0% among students with an irregular diet and poor self-reported health status, although this was not statistically significant.

In the bivariate logistic regression analyses, students with low levels of perceived interpersonal support were more than five times more likely to suffer from depression than those with higher levels of support (Table 3). Those who had poor quality of sleep were more than eight times more likely to have depression than those who had good quality of sleep (OR=8.67, 95% CI=2.37-31.73). Students with an irregular diet also had stronger association with depression (OR=2.44, 95% CI=1.00-5.93).

Based on the results of the bivariate logistic regression analyses, factors which showed a level of significance of less than 0.1 were selected to be included in the multivariate model (Table 4). With other factors adjusted, those with lower levels of perceived interpersonal support were 10 times more likely to have depression (OR=10.34, 95% CI=2.59-41.24). Compared with those with a mid-range household income, those with a household income of more than 5,000 thousand won were more likely to suffer from depression (OR=6.79, 95% CI=1.51-30.52). As with diet and the quality of sleep, although these factors were not statistically significant, their effect sizes were similar compared to those of the bivariate analysis.

## DISCUSSION

Our study shows that the mean CES-D score of medical students in a single medical school was 14.1±8.6 and that 37.1% of these students appeared depression. Our results also suggest that low levels of perceived interpersonal support increases the risk of depression by more than 10 times, and that having a higher household income does not necessarily diminish the risk of depression. We have also found some evidence that poor quality of sleep, an irregular diet, and a poor self-reported health status may be associated with depressive symptoms in medical students.

Over the past few decades there have been an increasing number of studies that have evaluated depression and the mental health of medical students. Overall, these studies have consistently suggested that levels of depression among medical students are higher than the general population and the students' age-matched peers [1-6]. Although it used a different scale to measure depression, a study of university students in Korea revealed that the prevalence of depression was higher among medical students compared to university students in other studies [30]. The mean CES-D score among medical students in our study was also higher than the mean score of Korean women aged 18 or above in the general population [31].

Evidence from several longitudinal studies has suggested that upon entering medical school, a student's emotional status is not significantly different from that of the general population, but levels of depression increases thereafter, implying that a certain dimension of the medical education process and environment may exert a negative effect on students' mental health [6, 32, 33].

The medical education process is very stressful, in that the level of academic pressure and the workload is frequently overwhelming. In many cases, this causes medical students to either deliberately or reluctantly stop themselves from socializing, developing personal relationships, and enjoying extracurricular activities [10]. In our study, students with lower levels of interpersonal support were 10 times more likely to have depression. This finding is supported by the results of numerous previous studies in which inadequate social activities resulted in a decrease in the psychological health of medical students [10, 16]. These studies also showed that, seeking social support was correlated with positive emotions in medical students [18], that stronger marital support predicted a lower rate of depression in medical students [19], and that students who thought that medical school interfered less with their social and personal lives were psychologically more stable [20].

Theoretically, perceived levels of interpersonal social support are considered to reflect one's ability to cope with challenges to one's mental and physical health by "buffering" the pathogenic effects of stress [17, 34]. Although recent stressful life events are known to be associated with psychological distress and depression, the level of correlation between the two factors is reported to be relatively low. It has been speculated that this is due to social support, which has the potential to mitigate the negative effects of stressful events. In one historic study, it was suggested that the number of stressful events and the degree of social support interact on depression [17]. Based on the evidence suggesting that stress levels among medical students are above normal, this may imply that adequate social support would be more effective in reducing psychological stress and depression in medical students compared to the general population [1].

In our study, it was somewhat surprising to find out that subjects with a mid-range of household income had a significantly lower risk of depression compared to those with higher household incomes. Similar results were found in a study in which the subjects with a mid-range per capita household income had a lower likelihood of internalizing symptoms compared to the group with the highest incomes [21]. Although numerous studies with adolescents have indicated a positive relationship between low socioeconomic status and depressive symptoms [22-24, 35], there have been studies in which a higher income level was associated with depression [21], or where the prevalence of depression was shown to be similar across household income levels [36]. The results of this study add to the body of evidence that suggests that a lower socioeconomic status may not necessarily predict depression.

Changes in health-related behaviors and lifestyle have been known to adversely affect the health of medical students [10]. In line with our results, many other studies have suggested that a decrease in the quality of sleep may occur as early as in the student's first year in medical school, and that this factor is associated with psychological distress among medical students [10-12]. A longitudinal study observed that insomnia among medical school students is a risk factor of subsequent clinical depression [37]. Substance abuse, including smoking and excessive alcohol consumption is a well known health issue among medical students. Although this was not evident in our study, the results of other studies have suggested that the increase in smoking and alcohol consumption among medical students has detrimental effects on their mental health [13-15]. However because these changes in health-related behaviors may also become manifest as a consequence or a component of depression, further study is required in order to reach a conclusion on their causal relationships.

The psychological distress experienced by medical students is not only detrimental to their personal well-being, but has been suggested to be associated with poor academic performance, dropping out of school, and suicide [1, 6]. It is also widely acknowledged that this psychological distress and poor health-related behaviors are not only apparent during medical school, but are persistent throughout the students' professional careers, having a negative impact on the quality of patient care they provide [1, 6-10, 38]. However, despite the relatively high level of depression among medical students, only a small number reportedly seek help or receive treatment [4, 9, 10, 39]. This may be due to lack of access to mental health services for students in medical schools, a lack of awareness of their mental health status, or a reluctance to seek help due to the stigma attached to mental health counseling [3, 4, 9, 10]. It is necessary that medical schools provide mental health services that students can access with confidentiality. It is also recommended that medical schools incorporate an education program about depression and stress management courses as a part of the regular curriculum. Systematic measures put in place to encourage social support and extracurricular activities should also be undertaken.

It should be noted that due to the relatively small number of participants, who were limited to first and second year students, and who comprised a convenience sample from a single, all-female university, the generalization of the study results is somewhat limited. Although it is generally acknowledged that the prevalence of depression is higher and the prevalence of substance abuse is lower in the female population [2, 3, 13, 32, 35, 40], there are studies which show no significant gender difference in terms of depression [6, 9]. Further study with both genders should be carried out in order to gain stronger evidence.

Although a self-evaluated questionnaire has the potential to cause measurement errors, we had tried to minimize such errors or bias by treating the responses anonymously, without collecting any information which would allow personal identification. Although our response rate was relatively high at 81.6%, not being able to draw comparisons between the responders and the non-responders remains as a limitation of our study. Another limitation is that the survey was carried at a single point out in time the beginning of a semester, when the level of academic stress is reported to be relatively low, resulting in the possibility that we may have underestimated the prevalence of depression [10]. We were not able to analyze factors which are specific to medical students, such as perceived stress levels and burn-out, along with other notable factors which are possibly associated with social support, such as the availability of other family members at home and the type of housing [35].

One other possible limitation is that the ISEL measures perceptions of the availability of support but not whether support was actually obtained. However, it has been previously reported that the perception of the availability of support is a more sensitive indicator than objective network measures, because the buffering qualities of social support are said to be cognitively mediated [17].

Finally, our cross sectional study design limits our ability to make statements about causal relationships. Several previous studies have indicated that degrees of psychological stress and depression change over the years in medical school. While some studies have suggested that the psychological stress levels of medical students increase over time, peaking in their graduation year [20, 39], other studies show that the deterioration of the psychological health of medical students starts as early as in their first year in medical school and peaks in their second year of medical school [6, 16, 32, 35]. The results of previous studies are inconclusive as to which time period during medical training is most stressful for the students, and results may differ under different cultural and educational circumstances. Longitudinal studies measuring depression levels at different points during the years spent at medical school as well as within a semester would be the next step in further research.

Despite these limitations, this study identifies modifiable risk factors of depression such as lifestyle factors and interpersonal social support using the ISEL, which is an objective measurement. Our results demonstrate that medical students have a relatively high level of depression, and that efforts should be made to encourage the availability of social support in order to promote mental health in medical students.

## Figures and Tables

**Table 1:**
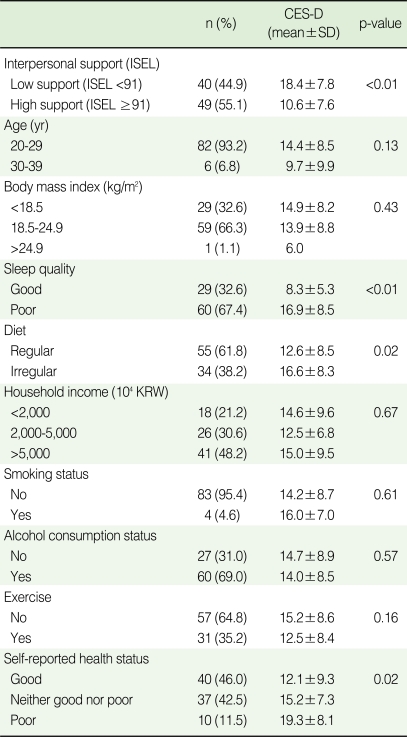
Center for epidemiologic studies depression scale (CES-D) scores according to social support, health-related behaviors, and socioeconomic status

Comparisons were made using the Wilcoxon rank-sum test and the Kruskal-Wallis test. ISEL, Interpersonal support evaluation list; KRW, Korea won.

**Table 2:**
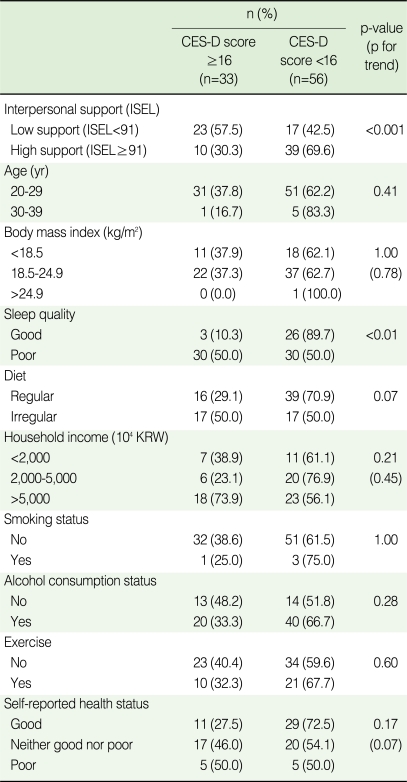
Prevalence of depression based on center for epidemiologic studies depression scale (CES-D) according to social support, health-related behaviors, and socioeconomic status

Comparisons were made using the Chi-square test, Fisher's exact test, and the Cochran-Armitage test for trends. ISEL, Interpersonal support evaluation list; KRW, Korea won.

**Table 3:**
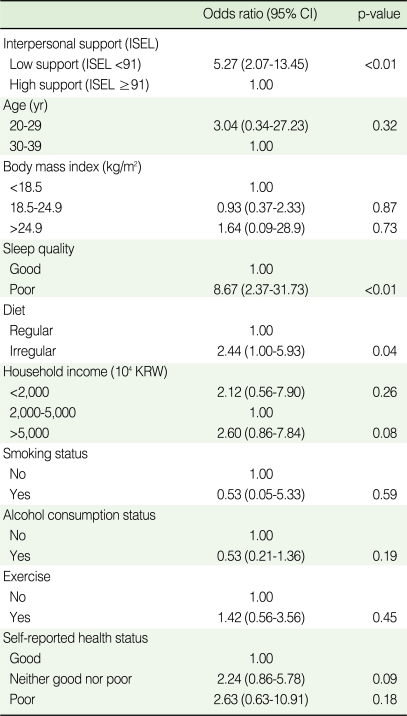
Bivariate logistic regression analysis for depression based on center for epidemiologic studies depression scale (CES-D) according to social support, health-related behaviors, and socioeconomic status

CI, confidence interval; ISEL, Interpersonal support evaluation list; KRW, Korea won.

**Table 4:**
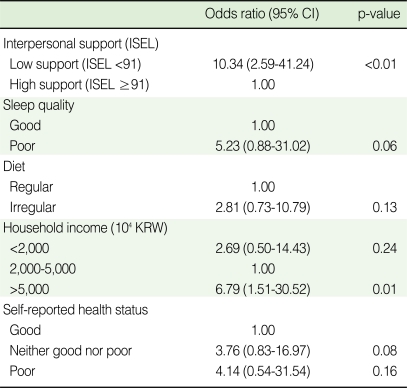
Multivariate logistic regression analysis for depression based on Center for epidemiologic studies depression scale (CES-D)

CI, confidence interval; ISEL, Interpersonal support evaluation list; KRW, Korea won.
